# Pattern of the First Recurrence Has No Impact on Long-Term Survival after Curative Intent Surgery for Perihilar Cholangiocarcinomas

**DOI:** 10.1155/2018/2546257

**Published:** 2018-08-09

**Authors:** Madalina Maria Blaga, Vladislav Brasoveanu, Cezar Stroescu, Mihnea Ionescu, Irinel Popescu, Traian Dumitrascu

**Affiliations:** ^1^“Carol Davila” University of Medicine and Pharmacy, Dionisie Lupu No. 37, 030167 Bucharest, Romania; ^2^Center of General Surgery and Liver Transplant, Fundeni Clinical Institute, Fundeni No. 258, 022328 Bucharest, Romania; ^3^“Titu Maiorescu” University, Gheorghe Petrascu No. 67A, 031593 Bucharest, Romania

## Abstract

**Aim:**

To explore the pattern of the first recurrence and impact on long-term survival after curative intent surgery for perihilar cholangiocarcinomas (PHC).

**Materials and Methods:**

Patients with curative intent surgery for PHC between 1996 and 2017 were analyzed. Survival times were estimated using the Kaplan-Meier method. Comparisons were made with the log-rank test.

**Results:**

A number of 139 patients were included. The median overall survival was 26 months. A recurrence was observed in 86 patients (61.9%), during a median follow-up time of 89 months. The median disease-free survival was 21 months with 1-, 3-, 5-, and 10-year estimated recurrence rates of 38%, 60%, 69%, and 77%, respectively. A number of 57 patients (41%) developed distant only recurrence, while 26 patients (18.7%) presented local and distant recurrences. An isolated local recurrence was observed in 3 patients (2.2%). The median overall survival was 15 months for patients with local recurrence, 15 months for patients with liver metastases, and 17 months for patients with peritoneal carcinomatosis (*p* = 0.903) as the first recurrence.

**Conclusion:**

Curative intent surgery for PHC is associated with high recurrence rates. Most patients will develop distant metastases, while an isolated local recurrence is uncommon. The pattern of recurrence does not appear to have a significant impact on survivals.

## 1. Introduction

Perihilar cholangiocarcinomas (PHC) are the most common variant of cholangiocarcinomas, and resection represents the single hope for a long-term survival [1].

For these patients, a curative intent surgery implies bile duct resection, locoregional lymph node dissection, caudate lobectomy, and usually a major liver resection [2]. Sometimes vascular resections are required to obtain negative resection margins [3].

This aggressive surgical approach was associated with significantly improved long-term survival rates at the expense of high morbidity rates [2], including clinically relevant complications [4]. Thus, a recent review has shown that in high-volume centers, the reported median overall survival time is 19–39 months, while the morbidity and mortality rates are 26%–75% and 0%–14.3%, respectively [2]. It appears that mortality rates are higher in Western series of patients, compared with Eastern series (13.6% versus 2.5%) albeit no significant differences of survivals were observed [5].

Despite improved survival rates with this aggressive approach, recurrence after curative intent surgery for PHC is a frequent event. Thus, the reported median disease-free survival times are 12–20 months [6]. Negative resection margins are of utmost importance for the disease-free survival [6, 7].

So far, there are only few studies exploring the pattern of the first recurrence after curative intent surgery for PHC [7–17]. However, the impact of the recurrence pattern on the long-term outcomes remains largely unknown. The knowledge of the pattern of the first recurrence and the impact on long-term outcomes after curative intent surgery for PHC might be of benefit for a better management of these patients.

The present study is aimed at exploring the pattern of the first recurrence and the impact on long-term outcomes after curative intent surgery for PHC, in a single center experience including a relatively large number of patients.

## 2. Materials and Methods

### 2.1. Patients

Between 1996 and 2017 (November 1st), a number of 150 patients underwent curative intent surgery for PHC, diagnosed at final pathology examination. A curative intent surgery was considered a surgical procedure associated either with negative resection margins or microscopic positive resection margins. Our criteria of resectability for PHC were described elsewhere [6].

Data were retrospectively reviewed from a prospective electronic database established in our institution.

### 2.2. Outcomes

The imaging follow-up of these patients included computed tomography and/or magnetic resonance imaging every 3 months during the first 2 years after resection and every 6 months after till 5 years after resection. Outside these dates, an imaging exploration was performed when the clinical suspicion of recurrence was raised. Recurrence was diagnosed either with imaging methods during the follow-up time or at relaparotomy for late complications. A local recurrence was defined as recurrence at the liver hilum (including hilar lymph node metastases), liver resection margin, distal common bile duct, or cholangiojejunostomy site. All other situations were considered distant metastases.

From the survival and follow-up, analyses excluded patients with postoperative mortality within 90 days (9 patients—6%) and those with no follow-up data (2 patients—1.3%). Thus, the study cohort included 139 patients with a median age of 59 years (range, 21–77 years) and with slightly male predominance (75 patients—54%). The surgical procedures and pathology data are presented in Tables 1 and 2. The median preoperative CA 19-9 serum level in the present cohort was 216 UI/ml (range, 1–12,000 UI/ml). A number of 72 patients (51.8%) underwent adjuvant chemotherapy.

### 2.3. Statistical Analyses

Statistical analyses were performed using the SPSS (Statistical Packages for Social Sciences) version 20.0 software (SPSS Inc., Chicago, IL). The Mann–Whitney *U* test was used to compare continuous data between the groups, while Fisher's exact test (two-tailed) was used for categorical data. The disease-free survival time was considered the time from resection to the time of first recurrence, while the overall survival time was considered the time from resection to death occurrence or last follow-up (January 1st, 2018). For patients with recurrent disease, time to recurrence was considered the time from resection to first recurrence. The median follow-up time was calculated using the reversed Kaplan-Meier method. The survival times were calculated using the Kaplan-Meier method and comparisons between the groups were made with the log-rank test. *p* values less than 0.05 were considered statistically significant.

## 3. Results

The median overall survival time for the entire cohort was 26 months (range, 2–205 months) with estimated 1-, 3-, 5-, and 10-year survival rates of 75%, 45%, 29%, and 15%, respectively.

**Figure 1:**
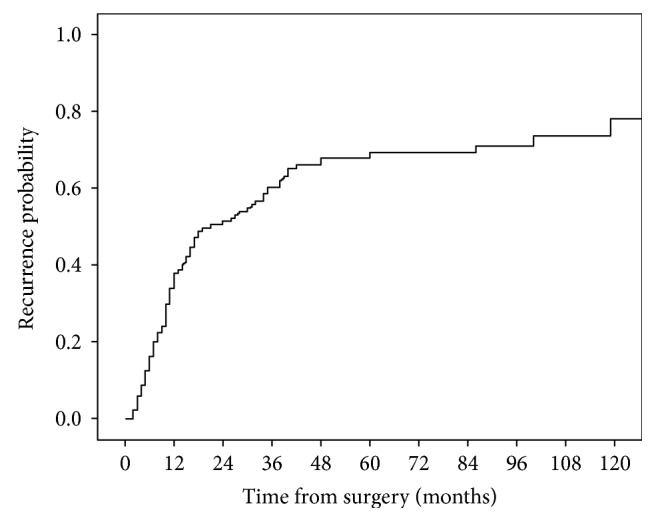
Estimated probability of recurrence after curative intent surgery for perihilar cholangiocarcinomas in 139 patients.

The median disease-free survival time for the entire cohort was 21 months (range, 2–205 months) with 1-, 3-, 5-, and 10-year estimated probability of recurrence rates of 38%, 60%, 69%, and 77%, respectively, as shown in Figure 1.

During follow-up time (median: 89 months, range: 2–205 months), a number of 100 patients (71.9%) died. In the group of death patients, the cause of death was related to tumor recurrence in 83 patients (83%), while 17 patients (17%) died of other causes, not related to tumor recurrence. Out of the 39 patients (29.1%) alive at follow-up point, 3 patients (2.1%) presented recurrence. Thus, a total number of 86 patients (61.9%) developed recurrence during the follow-up time in the present series.

Only one patient (0.7%) with local recurrence at distal common bile duct was amenable for reresection (i.e., pancreaticoduodenectomy), and his outcome was described elsewhere [18].

A number of 57 patients (41%) developed distant only recurrence, while 26 patients (18.7%) presented local and distant recurrences. An isolated local recurrence was observed in only 3 patients (2.2%), while a liver only recurrence and a peritoneal only recurrence were observed in 21 patients (15.1%) and 14 patients (10.1%), respectively. Overall, 29 patients (20.9%) presented local recurrence, 55 patients (39.6%) liver metastases, 40 patients (28.8%) peritoneal metastases, 6 patients (4.3%) retroperitoneal lymph node metastases, one patient (0.7%) lung metastases, and one patient (0.7%) ovarian metastases at the first recurrence.

In the group of patients with recurrent disease, the median time to recurrence was 11 months (range, 2–119 months), while the median survival time from recurrence to death was 4 months (range, 1–38 months). No significant differences of time to recurrence and time from recurrence to death were observed between patients with the first local recurrence, liver metastases, and peritoneal carcinomatosis (*p* value = 0.091, ns, data not shown).

**Figure 2:**
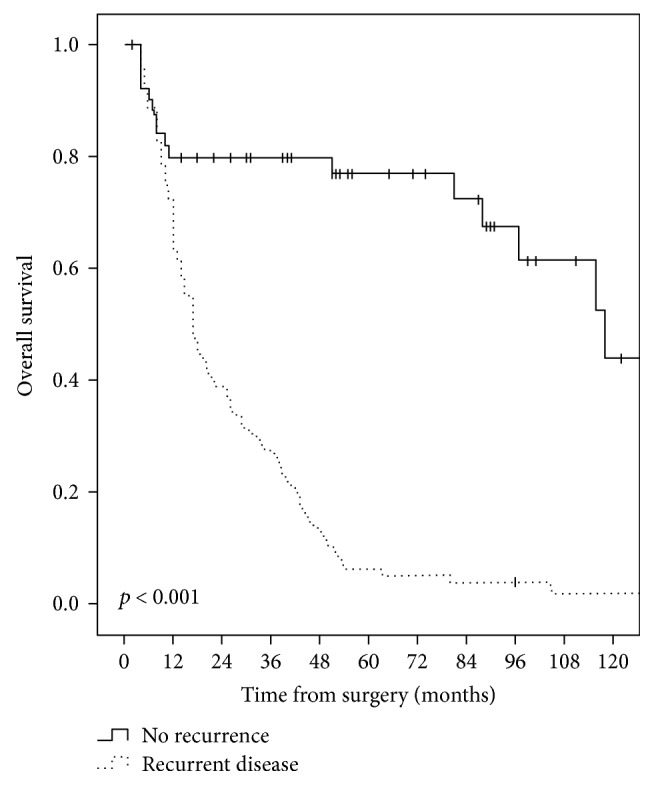
Comparative overall survival curves in patients with curative intent surgery for perihilar cholangiocarcinomas, with and without recurrent disease.

**Figure 3:**
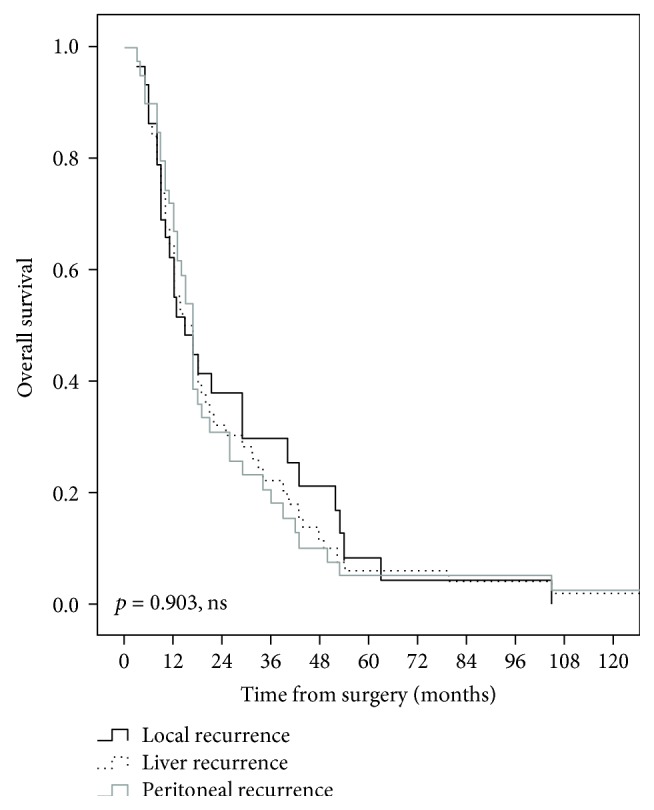
Comparative overall survival curves in patients with a different pattern of recurrence after curative intent surgery for perihilar cholangiocarcinomas.

**Figure 4:**
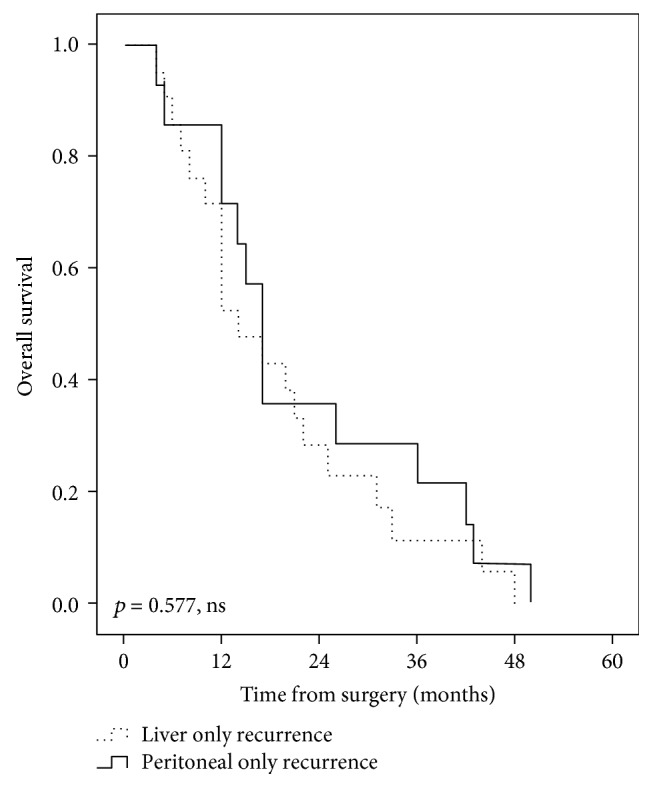
Comparative overall survival curves in patients with liver only recurrence and peritoneal only pattern of recurrence after curative intent surgery for perihilar cholangiocarcinomas.

The median overall survival time was significantly shorter in the group of patients with recurrent disease, compared with patients with no recurrence (17 months, range: 3–129 months versus 118 months, range: 2–205 months, *p* < 0.001), as shown in Figure 2. However, no statistically significant differences of survivals were observed between the groups of patients with a different pattern of recurrence (*p* = 0.903, ns), as shown in Figure 3. Thus, the median overall survival time was 15 months (range, 3–105 months) in the group of patients with local recurrence, 15 months (range, 3–129 months) in the group of patients with liver metastases, and 17 months (range, 3–129 months) for patients with peritoneal carcinomatosis at the first recurrence. Furthermore, no significant differences of survivals were observed between the groups of patients with liver only recurrence and peritoneal only recurrence (*p* = 0.472, ns), as shown in Figure 4.

It is worth mentioning that no significant differences were observed between the groups with isolated local recurrence, liver only recurrence, and peritoneal only recurrence with respect with the neutrophil-to-lymphocyte ratio (*p* = 0.691, ns), resection margin status (*p* = 1, ns), caudate lobe invasion (*p* = 0.564, ns), and adjuvant chemotherapy (*p* = 0.295, ns). The abovementioned factors were previously identified as independent predictors for disease-free survival after curative intent surgery for perihilar cholangiocarcinomas in our cohort of patients [6]. Furthermore, no differences between the groups were observed for age, gender, CA 19-9 serum level, tumor histology, pattern type, grade of differentiation and diameter, perineural invasion, and pTNM stages (*p* value = 0.076, ns, data not shown).

## 4. Discussion

The knowledge of the pattern of recurrence after curative intent surgery for PHC can be used for clinical decision-making [7, 9, 11, 12, 19]. Thus, for patients who are at high risk to develop distant recurrence, it was suggested that they are more likely to benefit from adjuvant chemotherapy [9, 12]. Recent studies have shown that adjuvant chemotherapy is an independent predictor for disease-free survival after curative intent surgery for PHC [6, 20]. For patients who are more likely to develop local recurrence, extensive surgery to obtain negative resection margins might be warranted [7, 9, 10]. Nevertheless, the follow-up after resection might be tailored to the recurrence pattern [9, 19].

Previous studies (Table 3) have shown an overall recurrence rate of 44%–68% after curative intent surgery for PHC [7–11, 13–15, 17, 21], during a median follow-up time of 18–102 months [7–11, 13–15, 17, 22], with 5-year recurrence rates of 67%–88% [7, 9, 15, 17]. In the present series, the overall recurrence rate was 61.9% during a median follow-up time of 89 months, with a 5-year recurrence rate of 69%.

Studies from literature have observed a local recurrence in 10.1%–26% of patients after curative intent surgery for PHC [7, 9, 14, 16], while distant metastases at the first recurrence were observed in 36%–45.8% of patients [7–11, 13, 14]. The most common site for distant recurrence was the liver in most studies [8–10, 14, 16], while peritoneum has been highlighted in few other studies [7]. An isolated local recurrence was observed only in 18%–19.1% of patients [7, 9]. In the present series, a local recurrence was observed in 20.9% of patients and distant metastases at the first recurrence in 59.7%, with liver as the most common site. Interestingly, in the present series, an isolated local recurrence was observed in only few patients (2.2%).

Recurrence as cause of death was observed in 91% of patients with curative intent surgery for PHC in the recent study [9] and has a detrimental effect on overall survival [13], as it was the case in the present series.

Several studies have shown a median time to the first recurrence of 12–31 months after curative intent surgery for PHC [9, 11, 14, 15, 21], while the median survival time from recurrence to death was 8–8.5 months [7, 9], with no significant differences between patients with local or distant recurrence [7, 9, 11, 14]. Similar findings were observed in the present series where the median time to the first recurrence was 11 months, with a median survival time from recurrence to death of only 4 months and no significant differences related to the pattern of recurrence.

It is worth mentioning that the recent study has shown that patients who developed an isolated local recurrence have had significantly better overall survivals, compared with patients who developed distant metastases with or without local recurrence (33.6 months versus 22.1–22.3 months, *p* = 0007) [17]. In the present cohort, no statistically significant differences of survivals were observed between the groups of patients with a different pattern of recurrence (*p* = 0.903, ns), as shown in Figure 3.

To date, there is no standard approach guidelines for recurrence after curative intent surgery for PHC [19]. For patients with isolated local recurrence, there is the potential benefit of reresection [7, 18] or radiation therapy [23], while for patients with distant metastases as the first recurrence, surgery has a limited place [8], except for some highly selected patients [24]. It appears that preoperative biliary drainage [25], associated vascular resections [3, 6, 26], and adjuvant chemotherapy [20] do not influence local or distant recurrence rates after curative intent surgery for PHC.

The recent study has shown that surgery for recurrent biliary tract cancer may prolong survival, but few patients are suitable for reresection [27]. In the present cohort, only one patient (0.7%) with local recurrence at the distal common bile duct was amenable for reresection. Nevertheless, there are studies that reported nil reresection rates for recurrences after curative intent surgery for PHC [8].

The present study has some limitations. First, it has a retrospective design. Furthermore, the imaging surveillance was heterogeneous (some patients have had computed tomography, and some other patients have had magnetic resonance imaging; the imaging exploration was performed in a large number of imaging centers with different expertise).

## 5. Conclusions

Recurrence after curative intent surgery for PHC is a common event. Furthermore, recurrence of disease is the most frequent cause of death in these patients. Most patients will develop distant metastases, while an isolated local recurrence is uncommon. Reresection has a limited role in treatment of patients with recurrence after resection for PHC. The pattern of recurrence does not appear to have a significant impact on overall survivals.

## Figures and Tables

**Table 1:** Operative procedures in 139 patients with curative intent surgery for perihilar cholangiocarcinomas.

Operative procedure	Number of patients (%)
Simple bile duct resection	24 patients (17.3%)
Right hemihepatectomy	37 patients (26.6%)
Right trisectionectomy	10 patients (7.2%)
Left hemihepatectomy	62 patients (44.6%)
Left trisectionectomy	1 patient (0.7%)
Central hepatectomy	1 patient (0.7%)
Caudate lobectomy	88 patients (63.3%)
Portal vein resection	22 patients (15.8%)
Hepatic artery resection	4 patients (2.9%)
Pancreaticoduodenectomy	1 patient (0.7%)

**Table 2:** Pathology data in 139 patients with curative intent surgery for perihilar cholangiocarcinomas.

Parameter	
Tumor diameter^†^, cm	2.5 (0.4–10)
Histology, adenocarcinoma	136 patients (97.8%)
Grade of differentiation	
G1	82 patients (59%)
G2	42 patients (30.2%)
G3	15 patients (10.8%)
Tumor pattern type	
Infiltrative	72 patients (51.8%)
Nodular	60 patients (43.2%)
Papillary	7 patients (5%)
pT stage	
T1	21 patients (15.1%)
T2	40 patients (28.8%)
T3	56 patients (40.3%)
T4	22 patients (15.8%)
Lymph node metastases (pN1)	54 patients (38.8%)
Distant metastases^∗^ (pM1)	9 patients (6.5%)
Perineural invasion	49 patients (35.2%)
Negative resection margins	105 patients (75.5%)

^†^Median value. ^∗^Liver metastases (8 patients) and celiac trunk lymph node metastasis (1 patient).

**Table 3:** Studies from the literature assessing the recurrence pattern after curative intent surgery for perihilar cholangiocarcinomas.

Author, year	Number of patients resected for PHC	Median follow-uptime (months)	Median disease-freesurvival (months)	Overall recurrence observed during follow-up time (%)	Recurrence rate at 5 years	Isolated local recurrence rate (%)	Distant metastases at the first recurrence (%)	Death observed during follow-uptime (%)	Recurrence as cause of death (% of total deaths)
Jarnagin et al. [11], 2003	76	24 months	NA	68%	NA	NA	36%	NA	NA
Ito et al. [10], 2008	38	29 months	31 months	65.8%	NA	NA	42.1%	NA	NA
Chen et al. [8], 2009	138	33 months	NA	49.3%	NA	NA	45.7%	NA	NA
Kobayashi et al. [14], 2010	79	30 months	NA	53%	NA	NA	43%	NA	NA
Saxena et al. [15], 2011	42	20 months	15 months	64%	88%	NA	NA	60%	NA
Wahab et al. [22], 2012	159	27 months	NA	59.1%	NA	NA	NA	NA	NA
Nuzzo et al. [21], 2012, multicentric	440	NA	NA	54.5%	NA	NA	NA	NA	NA
Groot et al. [9], 2015, multicentric	306	48 months	26 months	58%	67%	18%	40%	70%	91%
Kang et al. [13], 2016	260	102 months	NA	55%	NA	NA	39.7%	NA	NA
Komaya et al. [7], 2018	402	43 months	NA	61.7%	71.4%	19.1%	45.8%	64.9%	NA
Zhang et al. [17], 2018, multicentric	225	18 months	NA	44%	70.5%	NA	NA	NA	NA
Present series, 2018	139	89 months	21 months	61.9%	68%	2.2%	59.7%	71.9%	83%

## Data Availability

Data are available at our institution in an electronic database.
